# Oocyte vitrification modifies nucleolar remodeling and zygote kinetics-a sibling study

**DOI:** 10.1007/s10815-015-0446-x

**Published:** 2015-02-21

**Authors:** S. Chamayou, S. Romano, C. Alecci, G. Storaci, C. Ragolia, A. Palagiano, A. Guglielmino

**Affiliations:** 1 Unità Di Medicina della Riproduzione - Istituto HERA, via Barriera del Bosco n. 51/53, 95030 Sant’Agata Li Battiati, Catania Italy; 2 Dipartimento di scienze ginecologiche, ostetriche e della riproduzione, Seconda Università degli Studi di Napoli, Largo Madonna delle Grazie n. 1, 80138 Naples, Italy

**Keywords:** Embryo-kinetic, Oocyte, Time-lapse, Vitrification, Zygote

## Abstract

**Purpose:**

Oocyte vitrification does not affect embryo quality after oocyte warming, making this method effective in the preservation of female fertility. Morphokinetic parameters can be used to predict the competence of an embryo produced from fresh oocytes. Our aim was to study the effect of oocyte vitrification on zygote-embryo kinetics (pl).

**Methods:**

The embryo-kinetics of fresh and sibling vitrified/warmed oocytes were compared to determine the consequences of oocyte preservation on the timing of embryo development. A 44-hours time-lapse analysis, from the time of ICSI (t0), of 179 fertilized fresh oocytes was compared to 168 fertilized sibling vitrified/warmed oocytes.

**Results:**

Oocyte vitrification accelerated pronuclear disappearance, one-cell stage timing and modified nucleoli activity by increasing their number and decreasing their diameter at the zygote stage. In contrast, embryo kinetics during cleavage were similar to those observed for fresh sibling oocytes based on the parameters examined in this study.

**Conclusions:**

At the zygote stage, oocyte vitrification induces changes in pronuclei stability, probably due to pronuclei envelop instability as well as modifications in nucleoli functionality. Therefore, the predictive morphokinetic parameters on embryo competence found from fresh oocytes must be revised when applied on embryos from vitrified/warmed oocytes.

## Introduction

The aim of oocyte cryopreservation is to preserve oocyte integrity by exposure to very low temperatures thus arresting biological activity until clinical use. Oocyte freezing is effective for fertility preservation in oncological patients, for egg donation programs, or to delay pregnancy for medical or social reasons [21]. In Italy, this technology has been widespread for several years due to a severe regulation that prohibited the production and freezing of surplus embryos for each cycle of in vitro fertilization [24, 15].

The two methods used for oocyte cryopreservation are slow cooling followed by rapid thawing [12, 25, 4] and vitrification followed by rapid warming [19, 16]. The first method resulted in numerous pregnancies. However, the technical advances in vitrification, especially with the introduction of the Cryotop method, where the cell is immersed in a very small volume of cryoprotectant (0.1 μl), together with ultra-rapid cooling and thawing rates, made oocyte vitrification the widely preferred method due to higher oocyte survival [18, 23, 14, 3] and higher pregnancy rates, when compared to the slow freezing/rapid thawing method [7].

From the cellular point of view, we previously laid out that oocyte slow freezing/rapid thawing protocol induces a significant decrease in cleavage rate and embryo quality compared to sibling fresh oocytes [8]. On the contrary, a similar study have shown that fresh and sibling vitrified/warmed oocytes have similar embryo quality, cleavage and clinical pregnancy rates [26].

Oocyte cryopreservation has consequences at molecular level. The mRNA content is decreased by 60.6 % in slowly frozen/rapidly thawed oocytes and 36.7 % in vitrified/warmed oocytes when compared with fresh oocytes [9]. The decrease of mRNA is particularly relevant for mRNA of proteins involved in cell cycle regulation and processes, energetic pathway and DNA structural organization. These results gave an explanation of the differences of in vitro and clinical outcomes according to the method of oocyte cryopreservation.

Over the last few years, the introduction of time-lapse technology in the IVF laboratory has led to a multitude of studies that linked embryo-kinetics with the competence to develop and implant (see for review [29]). Recently, we proposed a method for the routine use of morphokinetic parameters to the aim of identifying embryos that have competence of developing and implanting within the first 3 days of in vitro culture [10].

In the present study, fresh and vitrified/warmed oocytes produced from the same ovarian stimulation (same patients), were processed for intracytoplasmic sperm injection (ICSI) and the embryos from both groups were cultured under identical conditions. For these reasons the kinetics of embryos produced from fresh and sibling vitrified/warmed oocytes are fully comparable to determine the consequences of oocyte cryopreservation on the timing of embryo development. The results will help understand whether informative morphokinetic parameters, previously determined on embryos produced from fresh oocytes, are also applicable for embryos produced from vitrified/warmed oocytes, or how they should be recalculated.

## Material and methods

### Population, ICSI, oocyte cryopreservation and embryo culture

The studied group is a mix of 47 patients aged between 29 and 39 years (mean age 34.1 years), with basal FSH between 2 and 11 IU/l (mean 6.4 IU/l) having undergone ICSI protocol after ovarian stimulation.

The ICSI technique has previously been described [8] and was performed as follows: All patients underwent ICSI treatment with fresh or vitrified/thawed oocytes and fresh ejaculated spermatozoa. Ovarian stimulation was achieved by the administration of luteal gonadotrophin-releasing hormone analogue (GnRHa) (Suprefact: Hoechst Marion Roussel Deutschland GmbH, Frankfurt, Germany) followed by recombinant FSH (Gonal-F: Merck-Serono, London, UK or Puregon, MSD, Franklin Lakes, USA) from cycle day 3. Vaginal ultrasound-guided aspiration of oocyte – cumulus complex was performed 35 h after human chorionic gonadotrophin administration (HCG 10,000 IU, Gonasi: AMSA, Rome, Italy). Oocyte denudation was performed 2 h after oocyte retrieval. ICSI on fresh oocytes was performed 1 h after oocyte denudation. After ICSI, in vitro culture was carried out in 25 μl of HTF cleavage Quinn’s medium (SAGE, Trumbull, USA) under mineral oil until day 3 (4–8 cells stage) in automated incubators with 5 % CO2, 5 % O2 at 37 °C, fitted with time-lapse imaging acquisition (Embryoscope, Unisense, Aarhus, Denmark). When embryo culture was prolonged after day 3, in vitro culture media was changed to HTF blastocyst Quinn’s medium (SAGE). During the incubation in the Embryoscope, five plane focal images were generated every 7 min and recorded. The kinetics of embryo development was recorded and reported for 44 h post-ICSI.

All patients gave written consent to freeze surplus metaphase II (MII) oocytes. The MII oocytes for freezing or fresh treatment were randomly chosen. The vitrification protocol used to freeze and thaw oocytes has been previously described [9]. Oocyte vitrification was started immediately after oocyte denudation. ICSI was performed 1 h after in vitro culture of vitrified/warmed oocytes.

For each couple, ICSI on fresh and vitrifed/warmed oocytes was performed using two fresh sperm samples from the male patient. The semen characteristics were similar in the two samples (sperm concentration, percentages of morphologically normal sperm and motile sperm). ICSI was performed with motile and morphologically normal spermatozoa.

### Definition of kinetic criteria

The definition and nomenclature of morphokinetic parameters recently published by a consensus of time-lapse user group were applied [13]. Time t0 was defined as the time of ICSI. Time t2PB was defined as the time of the completed second polar body detachment from the oolemma. Time tPNa was defined as the time of the first frame in which both pronuclei could be observed. Time tC was the frame with the last observation of both pronuclei. From this time, the nucleolus precursor bodies decreased in size and subsequently disappeared, followed by pronuclei membrane flattening. The successive frame, corresponding to one cell stage was annotated as tPNf. Finally, t2, t3 and t4 were defined as the times for the corresponding number of cells (t2 for 2 cells, etc.). The times were annotated at the first frame in which the cells (blastomeres) were seen as separated by individual membranes.

The difference tPNa-t2PB was the duration between the second polar body detachment and the appearance of both pronuclei. The difference tC-tPNa was the duration of time with both pronuclei (2PN) visualized. The difference tPNf-tC was the duration of pronuclei fading, in other words the time for complete nucleoli and then pronuclei envelop disappearance. The first round of cleavage was defined cc1 and it was calculated as t2-tPNf; the second round of cleavage was defined cc2 and it was calculated as t3-t2.

An additional parameter assessed was the synchronization of cell division(s), i.e. the time required for each blastomere to replicate and reach the successive cell cycle. The synchronization parameters were defined as s2 (calculated as t4-t3). All times were expressed in hours and fractions of an hour.

With a concern of data-homogeneity and because part of the embryos were transferred on day 2–3 and others on day 5, we decided to not include in the present study the morpho-kinetic parameters after t4 (day 2).

### Study of nucleoli

At tC, the number and diameter of nucleoli were calculated in both pronuclei in zygotes from fresh and vitrified/warmed oocytes. The nucleoli diameter was measured using Embryo-Viewer (Unisense).

### Statistical analysis

All analyzed data were continuous variables. The hypothesis about distributions of post-ICSI timings (t2PB, tPNa, tC, tPNf, t2, t3 and t4) and calculated timings (tPNa-t2PB, tC-tPNa, tPNf-tC, cc1, cc2, and s2) of embryos from two groups (embryos produced from fresh oocytes and embryos produced from vitrified/warmed oocytes) were tested by the two-sample Kolmogorov-Smirnov non-parametric tests. In both cases, each group was taken as an independent sample. Because of non-normal distribution, the medians were compared by Kruskall-Wallis non-parametric test.

The *Z*-test was used to establish different significance levels (*p* < 0.05; <0.001) between ratios.

All procedures were approved by the institutional review board of Unità di Medicina della Riproduzione – Istituto HERA.

## Results

### In vitro results

On fresh treatments, 253 MII oocytes were micro-injected. Surplus 270 MII oocytes were vitrified and warmed in a successive ICSI treatment. 239 of the warmed oocytes survived (survival rate: 88.5 %) and were micro-injected with fresh ejaculated partner sperm. In Table 1, in vitro results of ICSI on fresh and vitrified/warmed oocytes are detailed.

**Table 1:** In vitro results of ICSI with fresh and vitrifed/warmed oocytes

	Fresh oocytes	Vitrifed/warmed oocytes	*p*
n. cycles	47	47	–
n. micro-injected oocytes (%)	253	239	–
n. 2PN (% on micro-injected oocytes)	179 (70.8)	168 (70.3)	ns
2 cells-stage (% on 2PN)	179 (100)	168 (100)	ns
No multinucleation at 2C stage (% on 2C stage)	140 (78.2)	147 (87.5)	<0.05
1 multinucleated cell at 2C stage (% on 2C stage)	35 (19.6)	18 (10.7)	<0.05
Both multinucleated cells at 2C stage (% on 2C stage)	4 (2.2)	4 (2.4)	ns
2 even cells at 2C stage (% on 2C stage)	175 (97.8)	157 (93.5)	<0.05
2 uneven cells at 2C stage (% on 2C stage)	4 (2.2)	11 (6.5)	<0.05
3 cells-stage (% on 2C stage)	179 (100)	157 (93.5)	<0.001
4 cells-stage (% on 2C stage)	174 (97.2)	138 (87.9)	<0.001

The statistical significances between treatments with fresh and sibling vitrified/warmed oocytes (*p*) were compared with z-test

Results, in terms of fertilization and cleavage rates and the proportion of 2 multi-nucleated cells at the 2-cell stage were comparable in fresh and vitrified/warmed oocytes. The proportion of 2-cell stage embryos without multinucleated cells was higher in embryos from vitrified/warmed oocytes (78.5 % from fresh oocytes versus 87.5 % from vitrified/warmed oocytes, *p* < 0.05). The first cell division resulted in 2 even cells more frequently in embryos from fresh oocytes (97.8 % versus 93.5 %, *p* < 0.05). The percentage of 2-cell stage embryos reaching 3- and 4-cell stages were significantly inferior from vitrified/warmed oocytes (100 % versus 93.5 %; *p* < 0.001 and 97.2 % versus 87.9 %; *p* < 0.001 respectively).

### Kinetic results

The distribution of the timings t2PB, tPNa, tC, tPNf, t2, t3 and t4 and the calculated timings tPNa-t2PB, tC-tPNa, tPNf-tC, cc1 and cc2 were not normally distributed (Kolmogorov-Smirnov non parametric test*, p*-value < 0.05). The non-parametric Kruskall-Wallis showed no significant differences for the variable t2PB, tPNa and the calculated timing tPNa- t2PB, cc2 and s2 (*p*-value > 0.05), but significant differences (*p* < 0.05) for the variables tC, tPNf, t2, t3 and t4 and the calculated timings of tC-tPNa, tPNf-tC and cc1 were observed. Kinetic data are described in Table 2.

**Table 2:** Morphokinetic data from fresh and vitrified/warmed oocytes

	Fresh oocytes					Vitrified/warmed oocytes						
	n	Mean	DS	Median	Intervals	n	Mean	DS	Median	Intervals	*pKS* ^*1*^	*pKW* ^*1*^
t2PB	179	3.1	0.7	3.0	1.8;4.6	168	3.4	1.1	3.2	1.9;6.7	0.000	0.062
tPNa	179	7.7	1.5	7.5	4.7;11.2	168	8.3	2.5	7.6	4.6;14.2	0.000	0.125
tC	179	25.2	5.3	23.9	16.5;43.4	168	23.0	2.8	23.0	17.6;30.4	0.000	**0.001**
tPNf	179	26.1	5.8	24.6	17.0;46.8	168	23.6	2.8	23.4	18.1;30.7	0.000	**0.001**
t2	179	29.0	6.2	27.5	19.2;52.1	168	26.9	3.4	26.9	20.7;37.9	0.000	**0.007**
t3	179	39.4	6.9	38.6	23.6;60.1	157	37.1	5.1	37.4	25.2;52.7	0.006	**0.014**
t4	174	41.5	6.4	40.2	30.0;67.4	138	39.2	5.0	39.4	28.0;60.0	0.000	**0.002**
tPNa-tPB2	179	4.2	1.4	4.0	1.4;7.7	168	4.6	2.1	4.3	4.1;10.0	0.003	0.144
tC-tPNa	179	17.5	5.3	16.0	9.0;36.1	168	14.7	3.5	15.1	6.5;23.7	0.000	**0.000**
tPNf-tC	179	0.8	1.0	0.7	0.2;7.0	168	0.6	0.4	0.5	0.2;2.5	0.000	**0.038**
cc1(t2-tPNf)	179	3.5	3.5	2.7	1.3;25.1	168	3.3	1.7	2.8	2.0;13.2	0.000	**0.000**
cc2 (t3-t2)	179	10.3	4.5	11.9	0.0;17.7	157	10.6	4.6	12.0	0.0;20.2	0.000	0.460
s2 (t4-t3)	174	2.0	3.1	1.0	0.0;11.3	138	2.3	3.7	0.7	0.0;14.3	0.000	0.907

*pKS* ^*1*^ *p* value after Kolmogorov-Smirnov test, *pKW* ^*1*^ *p* value after Kruskall-Wallis test

**Fig. 1:**
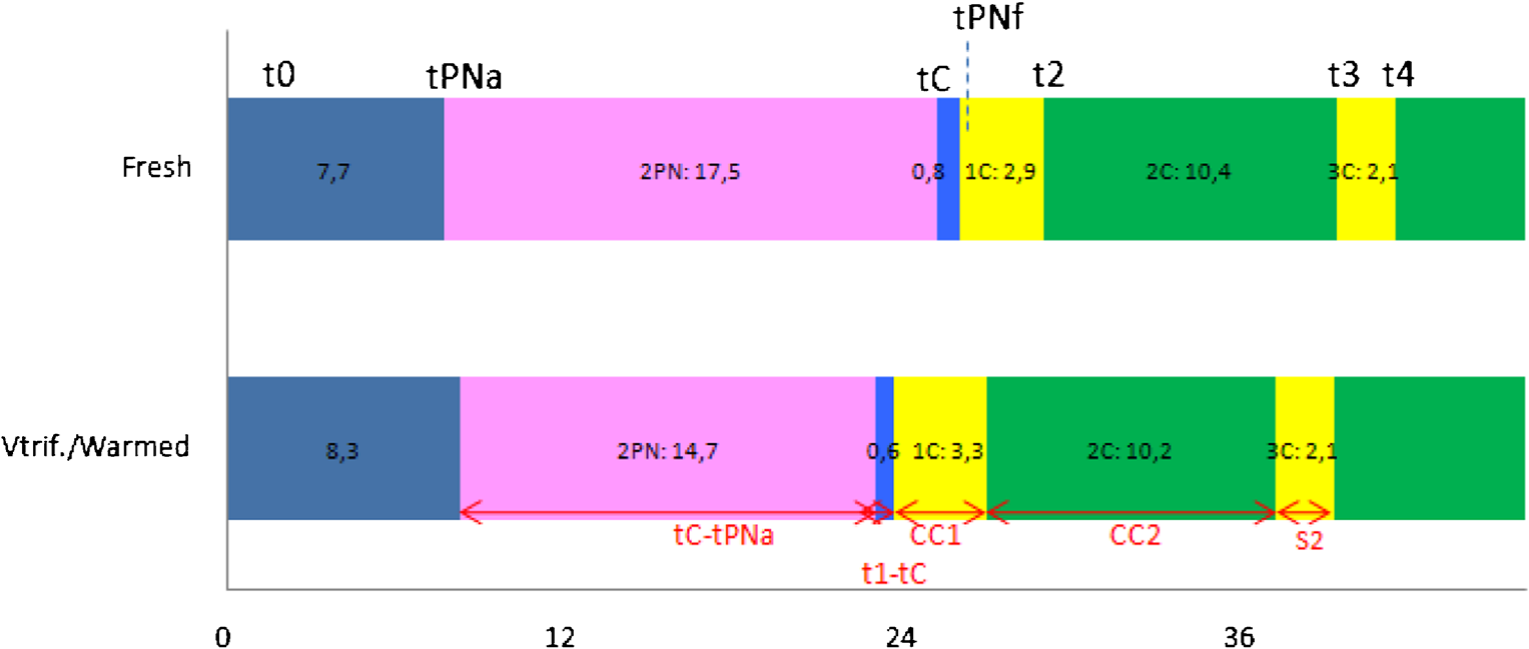
Embryokinetic from fresh oocytes and sibling vitrified/warmed oocytes from time 0 to 44 h

The morphokinetic parameters of zygotes and embryos from fresh and vitrified/warmed oocytes in the studied population are shown in Fig. 1.

### Nucleoli analysis

**Fig. 2:**
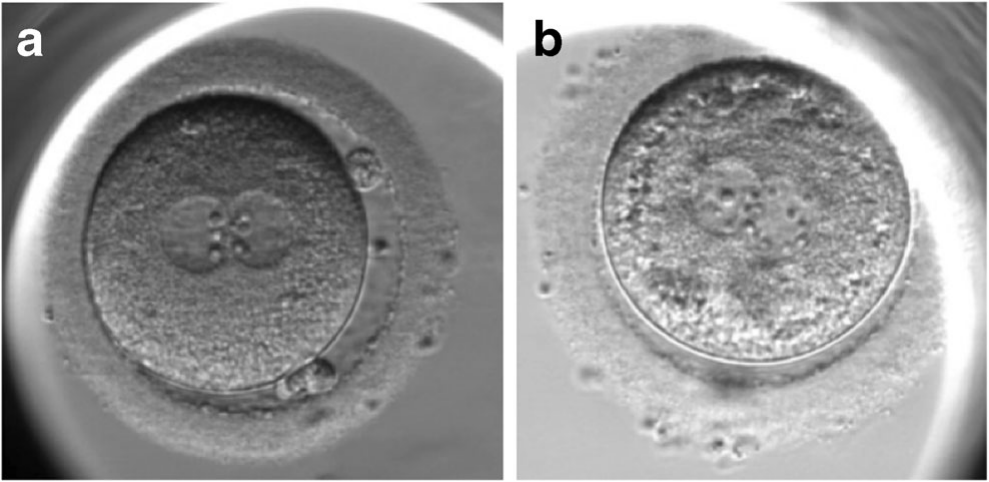
Nucleoli organization prior to pronuclei fading in zygote from fresh (**a**) and sibling vitrified/warmed (**b**) oocytes

At tC, the mean number of nucleoli was 4.1 [1;9] in pronuclei from fresh oocytes and 5.3 [1;11] in pronuclei from vitrified/warmed oocytes. The respective mean diameter of nucleoli was 3.1 and 2.6 μm in zygotes from fresh and vitrified/warmed oocytes. Figure 2 compares the nucleoli configuration in zygotes from fresh and vitrified/warmed oocytes.

## Discussion

From the present data, there is no difference in t2PB and tPNa between zygotes produced from fresh and sibling vitrified/warmed oocytes. Oocyte vitrification induced an anticipation of tC and tPNf in zygotes generated from vitrified/warmed oocytes compared to fresh sibling oocytes. The variations of these timings have as consequence the shortening of 2PN appearance (tC-tPNa) duration and cc1 in zygotes and embryos from vitrified/warmed oocytes. The timings t2, t3 and t4 are accelerated in the embryos generated from vitrified/warmed oocytes due to the shift of the previous phases (zygote stage). In the meantime, tPNa-t2PB, cc2 and s2 are not statistically different between embryos produced from fresh and sibling vitrified/warmed oocytes. In other words, it can be said that oocyte vitrification has no consequence on the completion of oocyte meiosis as demonstrated by the absence of variations of t2PB and the duration tPNa-t2PB between zygotes from fresh and sibling vitrified/warmed oocytes. On the contrary, oocyte vitrification has effects on the fertilization process and the first embryo mitosis events, as described by the decrease of the phases from the duration of observable 2PN until 2-cell stage. From 2-cell stage, the embryo kinetics are comparable in length and independent of oocyte source.

The shortening of tC-tPNa duration in vitrified/warmed oocytes could be due to pronuclei envelop instability (early evanescence), a problem of cytoskeleton traction (the pronuclei do not abut), or nucleoli functionality (nucleoli fade before agglomerating). From our data, we could not obtain information from the spatial organization of pronuclei within the zygotes measuring the distance at tC (just before PN fading) because two pronuclei were overlapped in 45.0 % of the zygotes and the distance between them could not be appreciated. By counting and measuring the diameter of nucleoli before tC, we found that the mean number of nucleoli was higher (5.3 versus 4.1) and their diameter was smaller (2.6 μm versus 3.1 μm) in zygotes from vitrified/warmed oocytes compared to zygotes from fresh oocytes, suggesting an early evanescence of nucleoli before they complete agglomeration in zygotes from cryopreserved oocytes. In respectively 17.9 % (32/179) and 25.6 % (43/168, *p* > 0.05) of the zygotes from fresh and vitrified/warmed oocytes, the difference of number of nucleoli between the two pronuclei and before PN fading was more than 2. According to Scott et al. [27], the presence of small scattered and unequal-sized nucleoli, as observed in zygotes from vitrified/warmed oocytes, could be indicative of functional defects with subsequent decreased and ineffective synthesis of rRNA. Recently, it has been demonstrated that the fusion of nucleoli is regulated by mitogen-activated protein kinase (MAPK) and maturation-promoting factor (MPF) [20]. The early nucleoli fading before nucleoli fusion could be due to MAPK and MPF activity dysregulation in zygotes from vitrified/warmed oocytes.

After sperm penetration, DNA synthesis starts synchronously within male and female pronuclei at about 12 h after sperm-oocyte fusion to reach the diploid one-cell stage at tPNf [5]. From our data, we cannot exclude that the DNA synthesis process would be disturbed by oocyte freezing because the modifications of kinetic are observed properly during DNA synthesis period (before tPNf). Further DNA analysis of arrested zygotes and embryos produced from vitrified/warmed oocytes could be informative.

Many articles have underlined the use of morphokinetic parameters for the determination of competent embryos [22, 11, 10, 17, 6]. In some studies, the very early morphokinetic parameters before cleavage stage have been determined as being informative. Azzarello et al. [2] used the time of pronuclei breakdown (corresponding here to tPNf) as a morphokinetic parameter predictive for embryo implantation. Aguilar et al. [1] defined the timings of second polar body extrusion, pronuclear fading and the duration S (corresponding here to tC-tPNa) as predictive factors of embryo competence. We previously determined tPNf and tC-tPNa to be informative on the day-3 embryo competence to become a viable blastocyst on day 5 of in vitro culture [10]. The present study shows for the first time how much a laboratory procedure such as oocyte vitrification can modify the morphokinetic parameters at zygote stage.

In a previous publication performed on fresh oocytes [10] we determined the morphokinetic parameters that are informative on the embryo competence to reach blastocyst stage on day 5 (IVD-MPK) and to implant when transferred (IMP-MKP). We also compared our results with those already published and underlined the necessity of each laboratory to determine its own informative parameters in its conditions of work. From the results reported here from vitrified/warmed oocytes, we observe an anticipation of tC and tPNf and all the successive timings even if cc2 and s2 remain unchanged. Under our working conditions, we should verify if the previous IVD-MKP and IMP-MKP remain valid on embryos produced from vitrified/warmed oocytes. We will have to take in consideration that the timing t2, t4, t7 and t8 could be shifted because of the anticipations of tC and tPNf. It will be interesting to observe how the previous determined ‘IVD-MKP’ tC-tF (corresponding here to tC-tPNa) would vary for competent embryos. In 2 embryos from vitrified/warmed oocytes that reached blastocyst stage on day 5 and implanted when transferred, the IVD-MKP tC-tPNa was shorter than the 7.7 h lower limit previously determined on embryos from fresh oocytes. All the other IVD-MKP and IMP-MKP were within the previously described ranges [10].

In the present study we show for the first time that oocyte vitrification has consequences on embryo kinetic at the zygote stage. Oocyte vitrification has no consequence on embryo kinetics at cleavage stage. The study of morphokinetic parameters is confirmed as a valid methodology for the determination of variation of embryo-kinetics generated by a procedure/protocol in the IVF laboratory. Furthermore, the retrospective time-lapse monitoring study gives the possibility of an in-depth analysis of a specific morphological trait such as nucleoli count and dimension before pronuclei fading.

In 2013, the practice committees of the ASRM and SART stated that oocyte vitrification and warming should no longer be considered experimental, and fertilization and pregnancy rates are similar to IVF/ICSI with fresh oocytes when vitrified/warmed oocytes are used as part of IVF/ICSI in young infertility patients and oocyte donor [28]. Nevertheless, the comparative study of fresh and sibling vitrified/warmed oocyte highlights that oocyte vitrification induces a moderate decrease of mRNA content [9] and a modification of zygote kinetic.
